# Preparation and evaluation of zeolites for ammonium removal from municipal wastewater through ion exchange process

**DOI:** 10.1038/s41598-020-69348-6

**Published:** 2020-07-24

**Authors:** Samuela Guida, Chris Potter, Bruce Jefferson, Ana Soares

**Affiliations:** 1 School of Water Sciences, Cranfield University, Cranfield, Bedfordshire MK43 0AL UK; 2 BYK Additives Ltd, Moorfield Road, Widnes, Cheshire WA8 3AA UK

**Keywords:** Environmental chemistry, Pollution remediation

## Abstract

The application of ion exchange process for ammonium (NH_4_^+^-N) removal from wastewater is limited due to the lack of suppliers of engineered zeolites which present high ammonium exchange capacity (AEC) and mechanical strength. This study focuses on the preparation and evaluation of synthetic zeolites (Zeolite1-6) by measuring AEC and resistance to attrition and compression, against natural (clinoptilolite) and engineered zeolite (reference, Zeolite-N). At high NH_4_^+^-N concentrations, Zeolite6 and Zeolite2 showed capacities of 4.7 and 4.5 meq NH_4_^+^-N/g media, respectively. In secondary effluent wastewater (initial NH_4_^+^-N of 0.7 meq NH_4_^+^-N/L), Zeolite1, 2 and 6 showed an AEC of 0.05 meq NH_4_^+^-N/g media, similar to Zeolite-N (0.06 meq NH_4_^+^-N /g media). Among the synthetic zeolites, Zeolite3 and 6 showed higher resistance to attrition (disintegration rate = 2.7, 4.1 NTU/h, respectively) when compared with Zeolite-N (disintegration rate = 13.2 NTU/h). Zeolite4 and 6 showed higher resistance to compression (11 N and 6 N, respectively). Due its properties, Zeolite6 was further tested in an ion exchange demonstration scale plant treating secondary effluent from a municipal wastewater treatment plant. However, Zeolite6 disintegrated after 2 months of operation, whilst Zeolite-N remained stable for 1.5 year. This highlighted the importance of the zeolite’s mechanical strength for successful application. In particular, future work should focus on the optimization of the zeolite production process (temperature, time and dimension of the kiln during calcination) to obtain an engineered zeolite with a spherical shape thus reducing eventual sharp edges which can affect mechanical strength.

## Introduction

**Figure 1:**
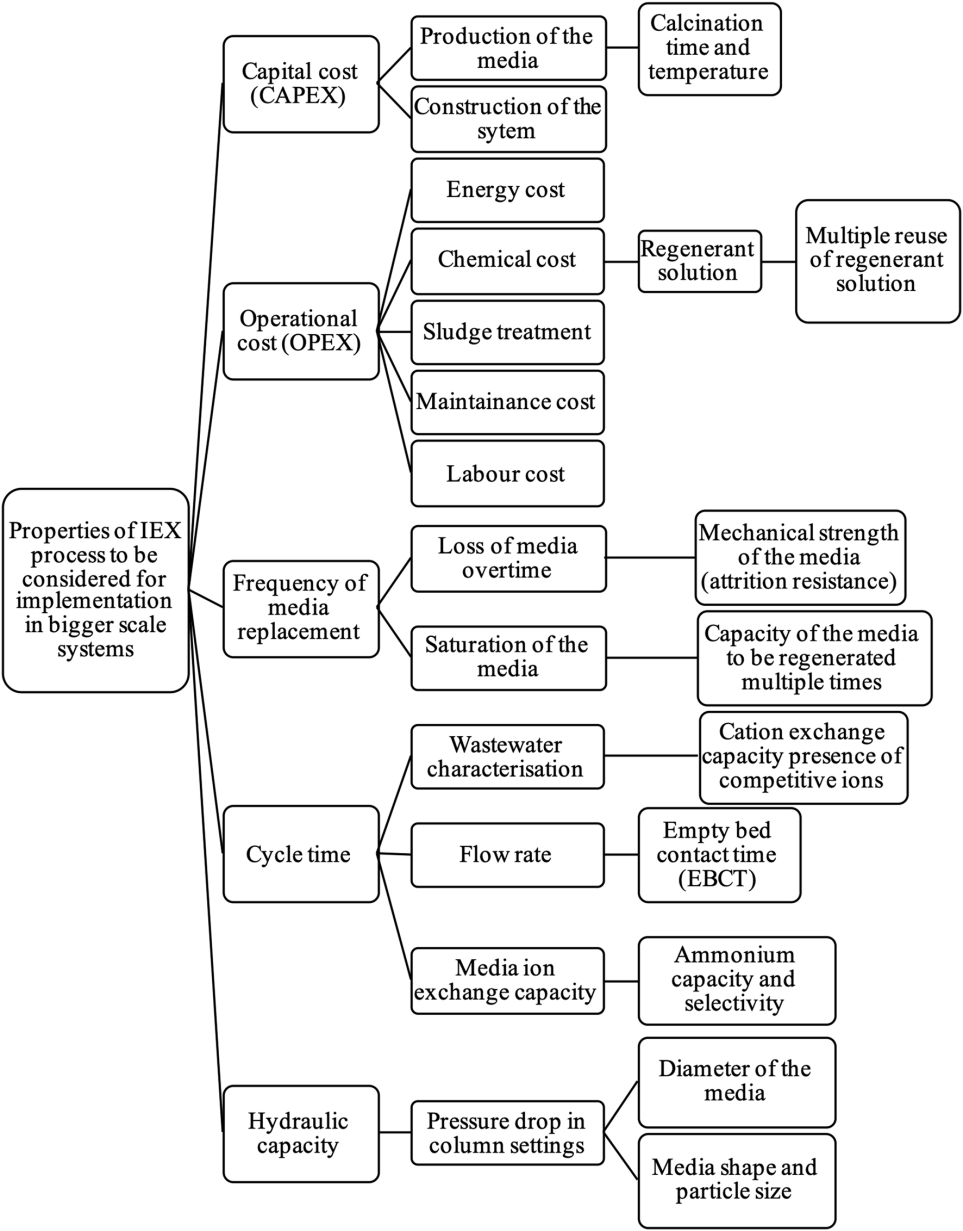
Properties of the ion exchange (IEX) process and characteristics of the IEX media needed to be considered for practical implementation of the IEX process at bigger scale.

Nitrogen compounds, such as ammonium (NH_4_^+^-N), nitrite (NO_2_^–^N) and nitrate, (NO_3_^–^N) can have a detrimental effect on the water quality of rivers and lakes^1^. For this reason, the Water Framework Directive 2000/60/EC and Council Directive 91/271/EEC have established stringent limits on the discharge of these compounds into water bodies^2,3^. These regulations require that the effluent of wastewater treatment plants have an ammonium concentration as low as 1 mg NH_4_^+^-N/L and maximum 30 and 50 mg NO_3_^–^N/L for nitrate discharge in freshwater and seawater, respectively^1^^–^^3^. Conventional methods to decrease ammonium concentration from wastewater, such as biological nitrification–denitrification^4^, present limitations obtaining such a low total nitrogen concentrations and environmental concerns due to the production of greenhouse gases^5^. The use of ion exchange (IEX) systems for the selective removal of ammonium from wastewater is becoming increasingly attractive due to its high removal efficiency, low greenhouse gas emissions, competitive cost, and relative simplicity of operation^6^. The most effective IEX media are characterized by great selectivity for the target pollutant, high specific surface area and ability to be regenerated allowing for its re-use multiple times^7^. However, most of the recent studies concerning the IEX process for ammonium removal are limited to laboratory scale analysis^1^. It is crucial to investigate the loss of ion exchange media and its annual replacement as this can increase costs significantly^8^. Other parameters that can impact costs are media capacity in between regenerations (more specifically the empty bed contact time, EBCT)^1^ and hydraulic capacity^9^. These properties can be correlated to the production of the IEX media and its physico-chemical characteristics such as the cation exchange capacity^10^, the selectivity for ammonium in presence of competitive ions^11^, as well as its mechanical strength (Fig. 1).

Currently, the most used ion exchange media for ammonium removal is the natural zeolite clinoptilolite (in the activated Na-form) which has been showed to have ammonium exchange capacity of up to 19 g NH_4_^+^-N/kg media (1.1 meq NH_4_^+^-N/g media) when treating domestic wastewater with initial concentration of 27 mg NH_4_^+^-N/L (1.5 meq NH_4_^+^-N/L) and pH 7.7^12^.

Zeolites are well known crystalline aluminosilicate minerals found in nature containing a framework of [SiO_4_]^4−^ and [AlO_4_]^5−^ tetrahedra linked by their corners through oxygen atoms. Natural zeolites are characterized by the general formula of M_2/n_OAl_2_O_3_xSiO_2_yH_2_O where *M* is the metal cation which compensates for the excess negative charge of the tetrahedra, *n* is the cation valence, *x* the number of Si tetrahedra (varying from 2 to 10) and *y* is the number of water molecules (varying from 2 to 7)^11,13^. The chemical bond between oxygen shared by the tetrahedra leads to the formation of cages and channels within the zeolite matrix, which can lodge water molecules as well as cations (usually Na^+^, Ca^2+^, K^+^ and Mg^2+^). These cations can be exchanged with the surroundings, following Eq. (1).

1 $${z}_{B}{A}^{{z}_{A}^{+}}+{z}_{A}B{Lz}_{B}\leftrightarrow {z}_{A}{B}^{{z}_{B}^{+}}+{z}_{B}AL{z}_{A}$$

where ${z}_{A}^{+}$ and ${z}_{B}^{+}$ are the valences of the respective cations and *L* is defined as the portion of the zeolite framework that is negatively charged^13^. In the presence of ammonium rich liquids, NH_4_^+^-N is exchanged with the cations present in the framework^14^. The exchange capacity of the zeolite depends on several factors such as the negative charge of its framework structure (due to Si/Al ratio) as well as size, concentration and charge of the exchange ions^13^.

To improve the selectivity and the exchange capacity, natural zeolites can be engineered by single or combined treatments that include heating and reaction with chemicals such as acids, bases and inorganic salts^15^. The chemical modification of clay and other aluminium-bearing minerals leads to formation of synthetic zeolites, such as Zeolite-N, which has an ammonium exchange capacity (AEC) up to 45–55 g NH_4_^+^-N/kg (2.5–3.1 meq NH_4_^+^-N/g media), that is significantly greater that the natural occurring zeolites^16,17^. The natural materials present octahedrally coordinated Al^3+^ while the synthetic zeolites presents tetrahedrally coordinated Al^3+^, which results in increased AEC^18^.

The scale-up of the IEX process using engineered zeolites for the removal of ammonium from wastewater is limited by the lack of commercial suppliers. Zeolite-N presented high potential for the wide application of IEX processes^18^, but it was commercially available for only a short period of time and no substitute media is currently on the market. Therefore, to maintain the operation and avoid decommissioning of already existing IEX plants as well as implement the process at bigger scale, it is a crucial need to find suitable media that can replace or even surpass the performance of Zeolite-N. However, to the authors knowledge, no study has been performed to investigate possible engineered zeolites for this application. Existing studies mainly focus on measuring or maximising exchange capacity and very few emphasise the mechanical strength of the media. The latter, is considered an essential characteristic to prevent cracking and disintegration of media in the IEX fixed bed columns which could result in the need to replace the media frequently as well as the need of additional filtering equipment^19^. The aim of this work was to test zeolites granulated through different calcination processes (temperature and time) and investigate its impact on the ammonium exchange capacity, regeneration efficiency and mechanical strength (resistance to attrition and compression), the key characteristics for IEX application by the wastewater industry.

## Results

### Maximum ammonium exchange capacity in mono-component solution

**Figure 2:**
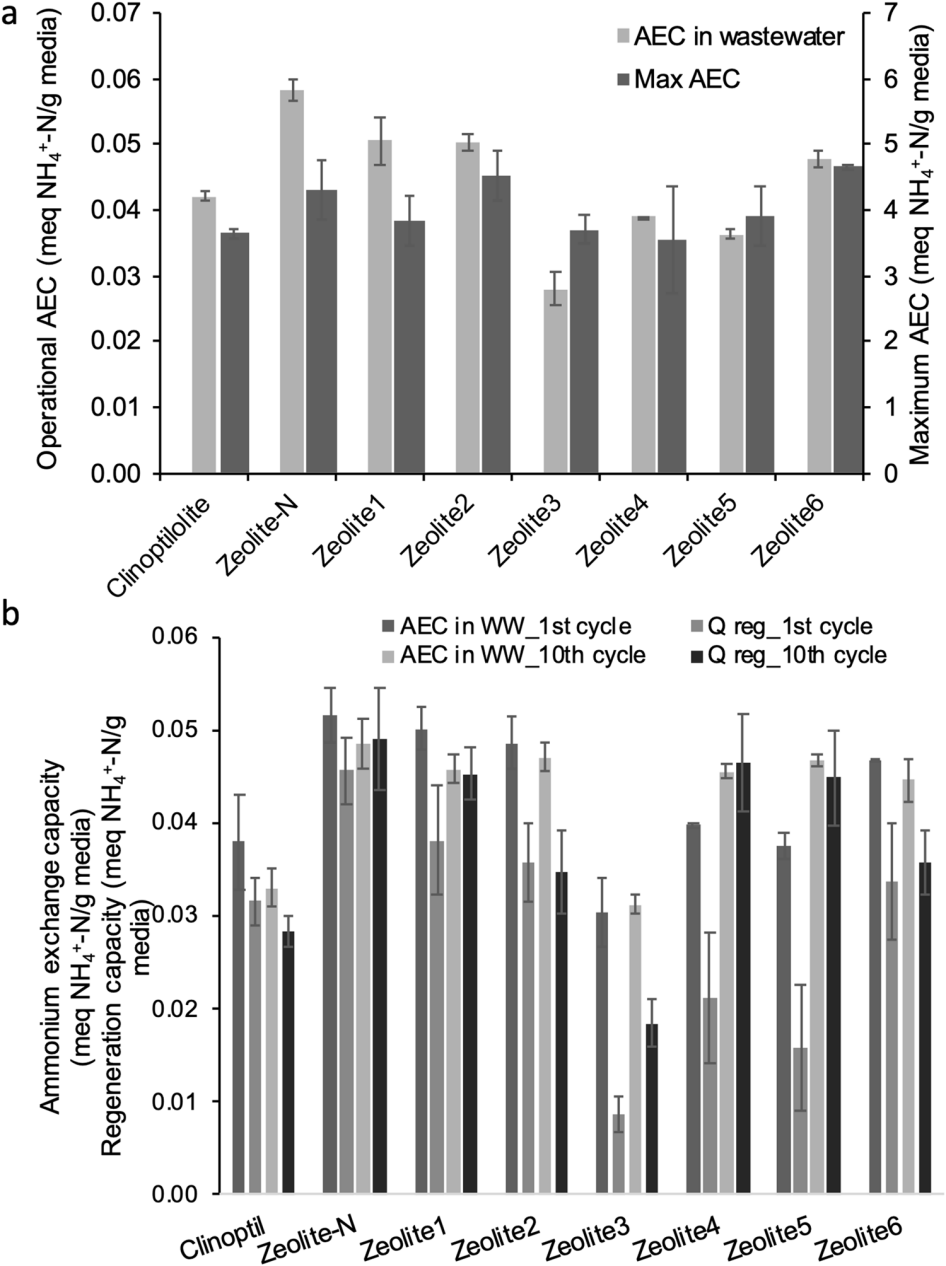
Operational ammonium exchange capacity (AEC) in municipal wastewater (C_in_ = 0.7 meq NH_4_^+^-N/L) and maximum AEC in mono-component solution (C_in_ = 55.6 meq NH_4_^+^-N/L) of natural and synthetic zeolites—(**a**); and comparison of the AEC in wastewater and regeneration capacities (Q_reg_) of the media at cycle 1 and cycle 10 of batch test in municipal wastewater (C_in_ = 0.7 meq NH_4_^+^-N/L)—(**b**).

When using a mono-component solution containing a high ammonium concentration (55.6 meq NH_4_^+^-N/L, 1,000 mg NH_4_^+^-N/L), the maximum ammonium exchange capacity (AEC) of Zeolite-N was 4.3 ± 0.5 meq NH_4_^+^-N/g media (Fig. 2a). In comparison, Zeolite6 and Zeolite2 presented higher AEC (4.7 ± 0.04 and 4.5 ± 0.4 meq NH_4_^+^-N/g media, respectively) while the AEC of the other media ranged between 3.6–3.9 meq NH_4_^+^-N/g media with the lowest value registered for Zeolite4 (3.6 ± 0.8 meq NH_4_^+^-N/g media) (Fig. 2a).

### Operational ammonium exchange capacity in municipal wastewater

To investigate the operational AEC of the zeolites, experiments were also performed in municipal wastewater (Fig. 2a) with initial ammonium concentration was 0.7 meq NH_4_^+^-N/L (12.7 mg NH_4_^+^-N/L). Zeolite-N presented the highest AEC (0.06 ± 0.002 meq NH_4_^+^-N/g media), followed by Zeolite1, 2 and 6 (0.05 ± 0.003 meq NH_4_^+^-N/g media). Zeolite3, 5 and Clinoptilolite presented lower AEC (0.03–0.04 meq NH_4_^+^-N/g media). By using the statistical software JMP, it was confirmed that AEC depended on the zeolite used (*p* < 0.0001).

The stability of AEC in wastewater and regeneration capacity (Q_reg_) was assessed over multiple cycles as an indicator of the reusability of the media (Fig. 2b). Zeolite-N presented the highest AEC (0.052 ± 0.03 meq NH_4_^+^-N/g media) with minimum decrease (6%) between the first and tenth cycle. Zeolite-N also presented the highest Q_reg_ (0.046 ± 0.04 meq NH_4_^+^-N/g media) which slightly increased (7%) between the first and the tenth cycles, reaching a regeneration efficiency of 90–100% (Fig. 2b). For clinoptilolite and Zeolite2, after 10 cycles, the AEC decreased (15% and 3%, respectively) and the Q_reg_ too (11% and 3%, respectively) while no clear pattern in the variation of AEC and Q_reg_ was identified for the other zeolites (Fig. 2b). However, no statistical difference was found when comparing the first and tenth cycles (*p* < 0.986) for the AEC of all the media except Zeolite5 (*p* < 0.008).

Considering all the media and cycles, statistical analysis revealed a significant effect for the ammonium exchange capacity in wastewater and during the regeneration phase depending on the media used (*p* < 0.0001). Additionally, Q_reg_ was affected by the number of cycles (*p* < 0.0012) and it depended on the media used (*p* < 0.0219).

### Zeolites’ mechanical strength

**Figure 3:**
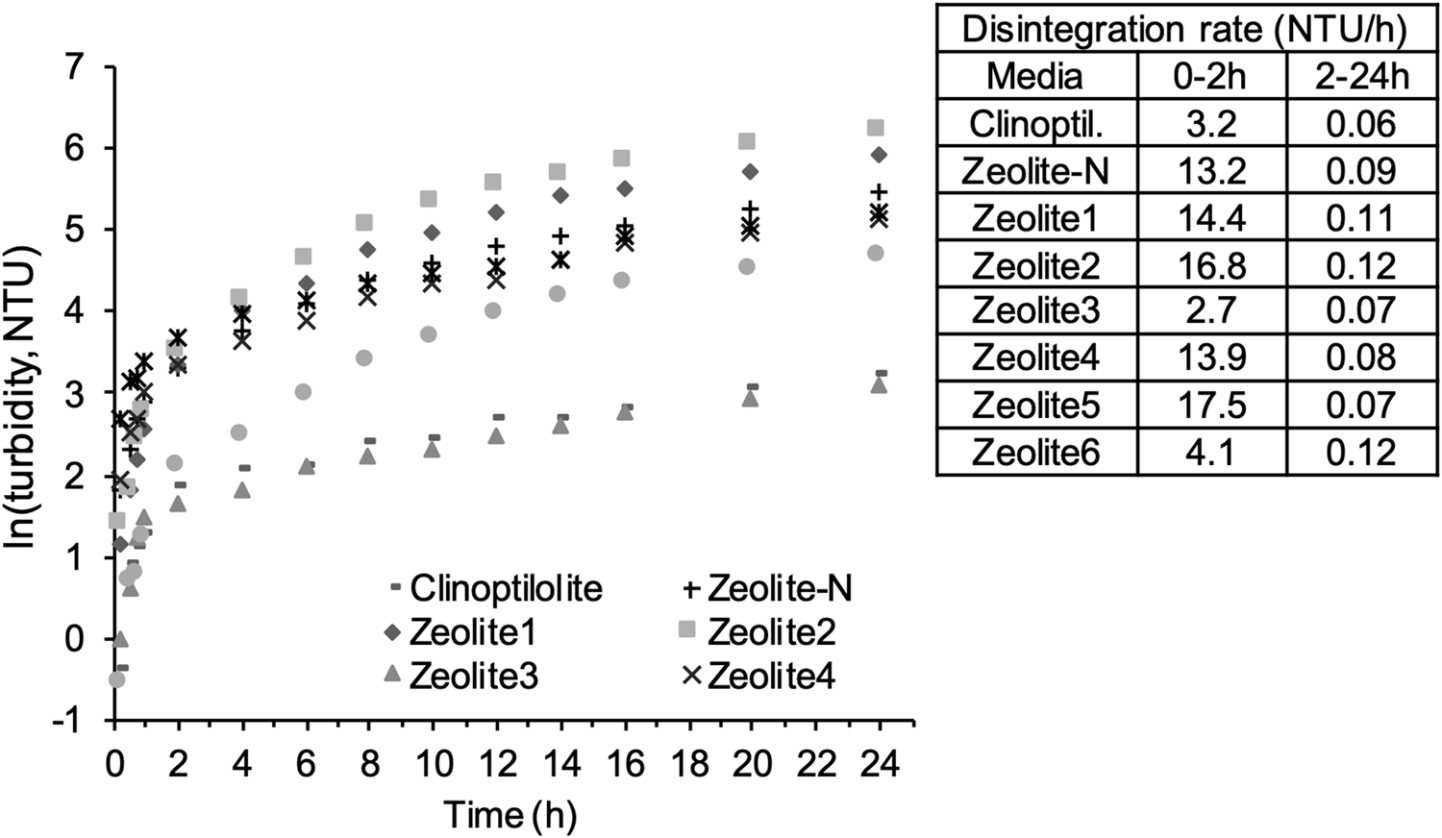
Turbidity measurements of the ion exchange media over 24 h of agitation at 200 rpm (t = 0-24 h), presented as ln(turbidity, NTU) over time, on the left, disintegration rate (NTU/h) of the ion exchange media in the period 0-2 h and 2-24 h.

The mechanical strength of the zeolites was defined as the media resistance to attrition and compression. The resistance to attrition was correlated with the turbidity measurements, following the principle that increase in turbidity was correlated to the media disintegration over time, as an indicator (Fig. 3). In the first 2 h of agitation, the disintegration rate of Zeolite-N was 13.2 NTU/h. In comparison, higher values were registered for Zeolite5 and Zeolite2 (17.5 and 16.2 NTU/h, respectively) and Zeolite1 and Zeolite4 (14.4 and 13.9 NTU/h, respectively). On the other hand, Zeolite3, Zeolite6 and Clinoptilolite showed high resistance to attrition with disintegration rates between 2.7 and 4.1 NTU/h. Lower disintegration rates were registered for all the media in between 2–24 h of agitation (0.09 ± 0.02NTU/h).

**Figure 4:**
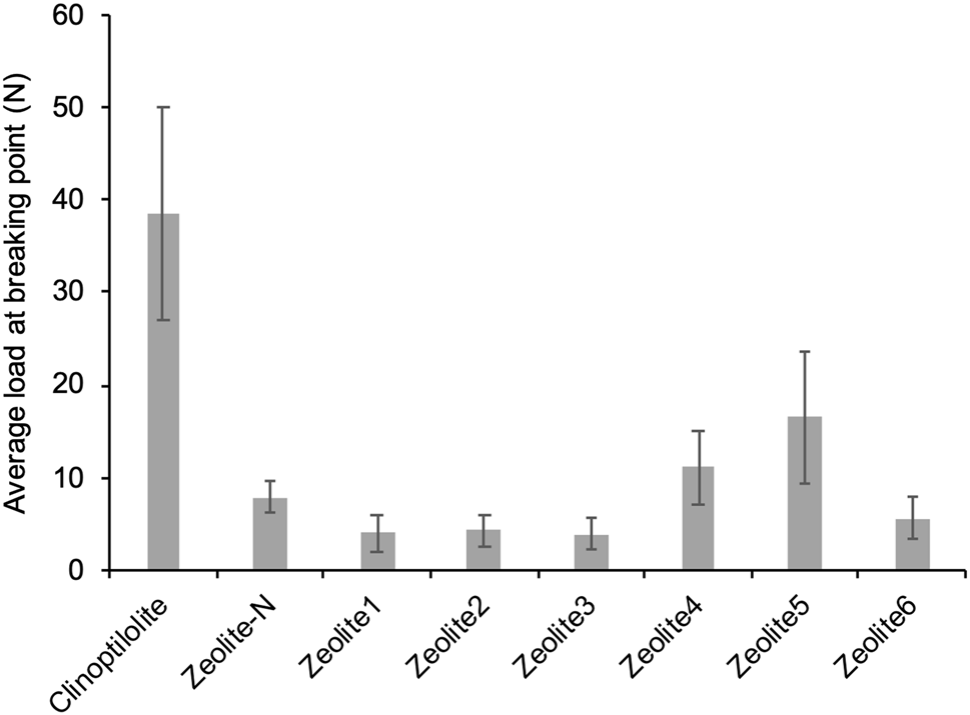
Maximum load at breaking point (N) registered for each media (average of 30 beads/media) when subjected to increasing pressure until breakage; force applied at breaking point was registered with an accuracy of 0.5%. Measurements were performed on fresh media.

For the compression test, each media (30 beads/media) was subjected to increasing loading pressure and the force applied at breaking point was registered (Fig. 4). Zeolite-N resisted to a pressure up to 7.9 ± 1.7 N before breakage. Clinoptilolite showed the highest compression resistance (loading pressure of 38.6 ± 11.5 N before breakage). Zeolite4 and 5 resisted a load up to 11.3 ± 3.9 N and 16.5 ± 3.2 N, respectively while, for the other media, the average load before breakage was between 3.9–5.7 N (Fig. 4).

### Implementation of zeolite at demonstration scale

**Figure 5:**
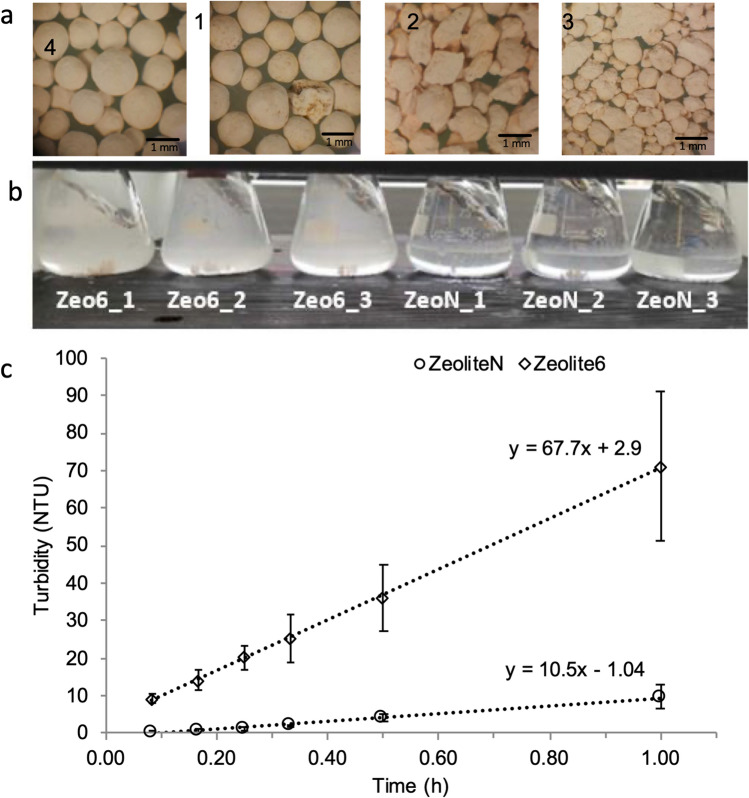
Optical microscope images (Optech Microscopes Ltd, 3X) of Zeolite-N (1; 2) and Zeolite6 (3;4) before (1;3) and after (2;4) operation of the IEX demonstration scale plant—(**a**); Erlenmeyer flask filled with Zeolite6 (Zeo6) and Zeolite-N (ZeoN) after the IEX demonstration plant operation, in agitation for t = 20 min, experiment in triplicate—(**b**); turbidity profile of Zeolite-N and Zeolite6 when subjected to agitation (at 200 rpm)—(**c**).

Zeolite-N and Zeolite6 were chosen among all the media tested for further tests in a demonstration scale IEX and fed with wastewater with an NH_4_^+^-N of 0.8 ± 0.3 meq NH_4_^+^-N/L (Table 2). In the IEX-D plant, Zeolite-N presented an AEC between 0.13–1.32 meq NH_4_^+^-N/g media and Q_reg_ between 0.05–0.29 meq NH_4_^+^-N/g media. On the other hand, lower values were registered for Zeolite6 for both AEC (0.01–0.95 meq NH_4_^+^-N /g media) and Q_reg_ (0.14–0.24 meq NH_4_^+^-N/g media). Zeolite6 was used for a total of 3 cycles due to unexpected intensive breakage of the media which clogged the media bed thus preventing the wastewater and regenerant to flow inside the IEX-D plant. In particular, a media loss of 0.027% per month was estimated for Zeolite-N, while, for Zeolite6, the loss was of 10% per month. After 7 and 3 cycles of operation with Zeolite-N and for Zeolite6, respectively, samples of each media were collected. Analysis at the optical microscope revealed high disintegration (smaller particles) for Zeolite6 when compared to the fresh material (Fig. 5a). When completing a strong agitation tests for a period of 1 h, Zeolite-6 showed higher disintegration rate (67.7 NTU/h) compared to Zeolite-N (10.5 NTU/h) thus indicating the lower mechanical strength (Fig. 5b,c).

## Discussion

Natural and synthetic zeolites were compared to the well-known Zeolite-N for their ability to remove ammonium (NH_4_^+^-N) as ion exchanging media from municipal wastewater (Table 1). The maximum ammonium exchange capacity (max AEC) in mono-component solution (C_in_ = 55.6 meq NH_4_^+^-N/L) was considered a primary good indicator for the comparison of the ion exchange media as it defines theoretical amount of ammonium ions that can be accommodated by the ion exchanger^20^. In accordance to literature^16,17^, Zeolite-N presented max AEC of 4.3 meq NH_4_^+^-N/g media. Similar results were obtained for Zeolite6 and Zeolite2 (4.7 and 4.5 meq NH_4_^+^-N/g media, respectively). The max AEC of the other media ranged between 3.6 and 3.9 meq NH_4_^+^-N/g media and, in particular, the max AEC obtained for Clinoptilolite (3.7 meq NH_4_^+^-N/g media) was in agreement with other studies^17^ (Fig. 2a). Other materials, such as hydrogels have been shown promise with capacities of 4.3 ± 0.5 mg NH_4_^+^-N/g in wastewater with initial concentration of 33 mg NH_4_^+^-N/L, contact time of 30 min and regeneration with pH 4 solution^21^.

**Table 1:**
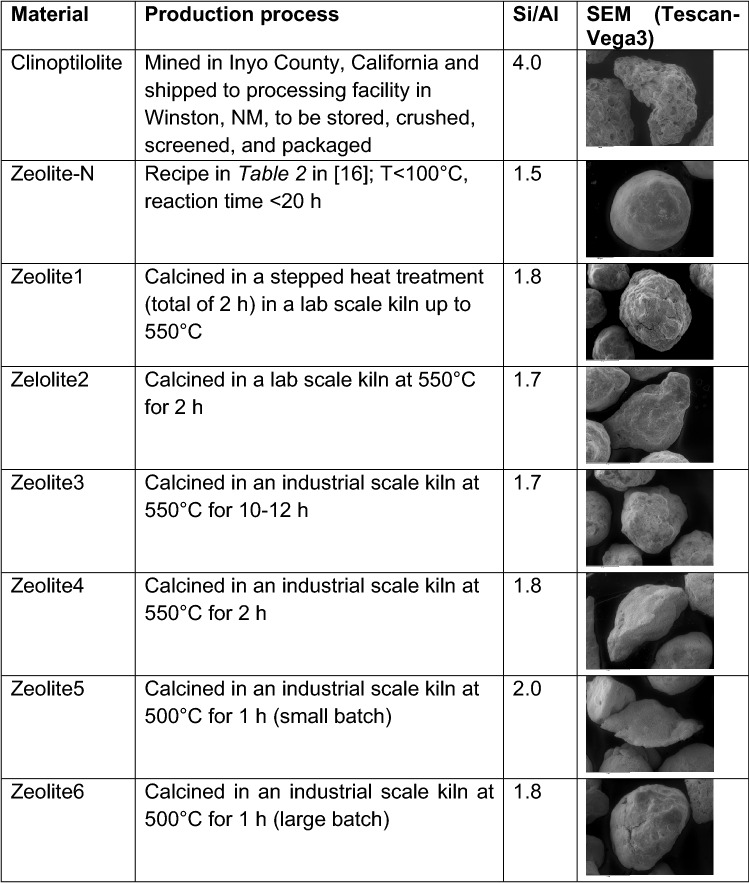
Natural and synthetic zeolites tested in this work.

**Table 2:** Ion exchange process—specification of the demonstration scale pilot plant.

	Units	Zeolite-N	Zeolite6
**Operational parameters**			
Max wastewater flow rate	L/day	10,000	10,000
Regenerant		10% KCl	10% KCl
Flow rate during normal operation	L/h	416.7	416.7
Flow rate during backwash	L/h	500	500
Volume column filled with media	L	69	35
Empty bed contact time (EBCT) during adsorption	min	10	5
Bed volumes of regenerant		10	10
**Column**			
Outer diameter	mm	315	225
Wall thickness	mm	4	4
Inner diameter	mm	307	217
Sectional area	dm^2^	7.4	3.7
Cylindrical column height	mm	1566	1577
Column volume	L	115.9	58.3

The AEC is dependent on the ammonium concentration in the solution^22^ and on the presence of competing ions (K^+^, Na^+^, Mg^2+^, Ca^2+^, Fe^2+;3+^) which can affect the selectivity for the ammonium ions^23^. In municipal wastewater (C_in_ = 0.7 meq NH_4_^+^-N/L), Zeolite-N presented the highest AEC both compared to the other synthetic zeolites and the natural clinoptilolite. The AEC in wastewater can be connected to the lower Si/Al ratio of Zeolite-N (Si/Al = 1.5) compared to the clinoptilolite (Si/Al = 4) and Zeolite1-6 (Si/Al = 1.7–2) (Table 1). In fact, an increase in the Si/Al ratio results in a decrease of the cation concentration and ion exchange capacity (proportional to the aluminium content)^24,25^. As for the maximum AEC, also in wastewater, similar results were obtained for Zeolite2 and 6 (Si/Al = 1.7 and 1.8, respectively), which indicated that these media could be chosen as potential candidates to replace Zeolite-N (Fig. 2a). The mechanisms of ammonia removal with zeolites have been indicated to occur via ion exchange and electrostatic interactions^26^. The latter is determined by the point of zero charge and isoelectric point that is the pH at which the zeta potential is zero, indicating a balance of positive and negative charges^27^. Clinoptilolite has been shown to have an isoelectric point < 2, meaning that this media is negatively charged at the wastewater pH and hence the ammonium ion is positively charged, there could be surface adsorption^28^. Nevertheless, with the zeolites tested in this study, the ammonia exchange capacity correlates well with the changes in Al:Si ratio and so this indicates that the ion exchange mechanism is potentially the more important removal pathway. Also, the fact the zeolites can be regenerated with KCl, also indicates the IEX pathway is dominant in relation to the electrostatic uptake. This was also the conclusion from the study from Sánchez-Hernández et al*.*^26^ using zeolite obtained from a hazardous Al-containing waste and NaCl as a regenerant.

When subjected to multiple cycles of ammonium uptake and regeneration, Zeolite-N presented similar values for AEC in wastewater and Q_reg_ thus indicating an efficient ion exchange process (Fig. 2b). When compared to Zeolite-N, Zeolite1-2 and 6, presented similar AEC which slightly decreased between the first and the last cycle. However, the Q_reg_ were lower when compared to the one of Zeolite-N indicating that the reusability of these media is not as high as Zeolite-N.

Successively, the media were compared for their mechanical strength. This property was in fact considered necessary for the use of the media in IEX columns where it is subjected to mechanical stress^19^. The strength of the media was studied in terms of resistance to attrition and compression (Fig. 3, 4). Clinoptilolite presented higher resistance to attrition and compression when compared to Zeolite-N. On the other hand, considering the synthetic zeolites, it was no possible to clearly conclude what media was stronger than Zeolite-N. In fact, Zeolite3 and 6 showed higher resistance to attrition, while Zeolite4 and 5 showed higher resistance to compression. It is important to consider that, as presented in Table 1, the media differed for shape and, in the case of the synthetic zeolites, for the production process. In particular, Zeolite1-6 were subjected to different calcination times and temperatures and calcined in different scales of kiln. According to Johnson et al*.*^29^ a prolonged calcination process, leaves the material completely porous, thus affecting its resilience or flexibility. In fact, Zeolite3, which was produced with the longest time of calcination when compared to the other media (12 h, industrial scale kiln), showed the lowest resistance to compression (3.9 N). Additionally, when compared to Zeolite-N, all the media presented a low sphericity, with sharp edges on their surface which could have had in impact on how the media responded to the mechanical stresses. Studies completed on the compressibility of various commercial granular activated carbon materials, also discussed the impact of media sharp edges and variability of the compressibility measurements^30^. These results suggested that particular attention needs to be given to the production of the media especially considering its porosity and shape. When producing new media, it is important to obtain an optimal size of the particles. In fact, the AEC of the media is also affected by the particle size with smaller dimension resulting in higher AEC due to an increase in specific surface area available for the ammonium exchange^6^. Following previous studies^1^, all the synthetic zeolites were filtered to obtain a 1–2 mm particle size. On the other hand, the size of the Clinoptilolite particles ranged between 2.5–3.5 mm which could have affected both the AEC capacity tests and the attrition and compression tests.

Finally, the possibility to replace Zeolite-N with one of the other media in a demonstration scale system was investigated (Table 2). In particular, Zeolite6 was chosen as, from the experiments conducted at laboratory scale, Zeolite6 and Zeolite-N presented similar ammonium exchange capacities in wastewater (0.06 and 0.05 meq NH_4_^+^-N/g media, respectively). Regarding the mechanical strength, the laboratory scale experiments, showed the Zeolite-N has slightly higher resistance to compression but lower resistance to attrition, when compared to Zeolite6. Following the results from previous studies^1,31^, at demonstration scale, Zeolite-N was used to remove ammonium from secondary effluent wastewater at an empty bed contact time of 10 min while 10 bed volumes of regenerant were used for restoring the initial capacity of the media. In these conditions, Zeolite-N presented AEC = 0.13–1.32 meq NH_4_^+^-N/g media and Q_reg_ = 0.05–0.29 meq NH_4_^+^-N/g media. When Zeolite-N was substituted with Zeolite6, a lower EBCT (5 min) was used to test the ammonium exchange capacity in wastewater of the media. Compared to Zeolite-N, Zeolite6 presented lower values for both AEC in wastewater (0.01–0.95 meq NH_4_^+^-N /g media) and during regeneration (0.14–0.24 meq NH_4_^+^-N/g media). The AEC capacity at higher EBCT could not be tested due to the intensive breakage of Zeolite6 forced the stopping of the column (10% media loss/month). The high disintegration of Zeolite6 was correlated to the low sphericity of the media when compared to Zeolite-N.

The ion exchange process is efficient at selectively removing ammonium from wastewater. The work completed demonstrates the potential benefit of using zeolite N and outlines the key challenges that any new zeolite N media must meet to be able to be used within the wastewater sector. The implementation ion exchange process at scale is limited by the lack of a supplier for the Zeolite N, which, as confirmed in this study, it is the most efficient ion exchange media for ammonium removal. Even though the high ammonium exchange capacity is a crucial property of ion exchange media, this work highlighted the necessity to investigate a more efficient production method, which can ensure high mechanical strength and, therefore, a longer lifespan of the media thus reducing the costs connected to a frequent media replacement.

## Conclusions

This study focused on the preparation and evaluation of synthetic zeolites (Zeolite1-6) by measuring AEC and resistance to attrition and compression, against natural (clinoptilolite) and engineered zeolite (reference, Zeolite-N). Finding an ion exchange media, which could substitute Zeolite-N, that is no longer available commercially, is important to ensure implementation of the process in wastewater treatment plants. The following conclusion were made:

1. Zeolite-N presented maximum AEC of 4.3 meq NH_4_^+^-N/g media. Zeolite6 and Zeolite2 showed increased capacities (4.7 and 4.5 meq NH_4_^+^-N, respectively).
2. In secondary effluent wastewater (C_in_ = 0.7 meq NH4 + -/L), Zeolite1, 2 and 6 showed AEC = 0.05 meq NH_4_^+^-N /g media, similar to the AEC registered for Zeolite-N (0.06 meq NH_4_^+^-N/g media).
3. All the media showed lower reusability during the 10 cycles of batch test when compared to Zeolite-N.
4. The natural clinoptilolite showed higher mechanical strength than Zeolite-N. On the other hand, among the synthetic zeolites, Zeolite3 and 6 showed higher resistance to attrition. Zeolite4 and 6 showed higher resistance to compression (11 N and 6 N, respectively). The variability of the mechanical strength of the synthetic zeolites was attributed to the difference in the production process (temperature, time and dimension of the kiln during calcination).
5. From the laboratory scale experiments, Zeolite6 was chosen as media to replace Zeolite-N at demonstration scale. However, once implemented, the media disintegrated after 2 months of operation (10% media loss/month).
6. This study highlighted the importance of producing synthetic zeolites with mechanical strength. In particular, future work should focus on the production of a media with more spherical shape thus reducing eventual sharp edges which could have an impact on both the resistance to attrition and compression.

## Material and methods

### Natural and synthetic zeolites

Zeolite-N (synthetic zeolite, NanoChem Pty Ltd, Australia) was used as reference material and compared to clinoptilolite (natural zeolite, St. Cloud Mining, New Mexico), and Zeolite1-6 (synthetic zeolites, BYK Additives & Instruments Ltd, Germany). The synthetic zeolites were produced according to Mackinnon, Millar and Stolz^16^, granulated according to procedure described in Table 1. Clinoptilolite had a Si/Al = 4.0 (natural zeolites were reported to have a Si/Al = 3–5^11,32,33^) while lower values of Si/Al (1.5–2.0) were measured for the synthetic zeolites (Table 1).

The zeolites were initially washed with deionized water to remove any dust from their surface and sieved to obtain the required size (1–2 mm for the synthetic zeolites and 2.5–3.5 mm for the clinoptilolite) before further tests.

### Municipal wastewater characterization

Municipal wastewater was obtained from Cranfield University wastewater treatment plant in the UK (2,840 population equivalent) after the secondary treatment with trickling filters that removed organic carbon. The wastewater was filtered (filter pore diameter 1.2 µm) to prevent any residual solids to interfere with the adsorption tests^34^. The composition of the wastewater was: chemical oxygen demand (COD) was 37.0 ± 12.3 mg/L, ammonium (NH_4_^+^-N) was 13.6 ± 4.6 mg was NH_4_-N/L, orthophosphate (PO_4_-P) was 6.0 ± 0.25 mg PO_4_-P/L, calcium (Ca^2+^) was 25.1 ± 1.2 mg Ca^2+^/L, potassium (K^+^) was 25.4 ± 2.2 mg K^+^/L and the pH was 7.3 ± 0.4.

### Ammonium exchange capacity (AEC) of zeolites

The maximum ammonium exchange capacity (AEC) of the zeolites was calculated in mono-component solution with an initial concentration of 1,000 mg NH_4_^+^-N/L (55.6 meq NH_4_^+^-N/L). Successively, the operational AEC in municipal wastewater was calculated starting from an initial concentration of 12.7 mg NH_4_^+^-N/L (0.7 meq NH_4_^+^-N/L).

The solutions (100 ml) were mixed with 0.5 g of media at 150 rpm for 8 h using the orbital shaker SSL1 (STUART, UK), after which, the remaining ammonium was measured. Experiments were conducted in triplicate. The AEC was calculated according to Eq. (2)^35^:

2 $$AEC=\frac{\left[{C}_{i}-{C}_{f}\right]*{V}_{ treated}}{M}$$

where *AEC* is the ammonium exchange capacity (meq NH_4_^+^-N/g media), *C*_*i*_ and *C*_*f*_ are the initial and final ammonium concentration in solutions (meq NH_4_^+^-N/L), *V*_*treated*_ is the volume of the solution (L) and *M* is the mass of media (g).

To investigate the reusability of the media over multiple cycles, the zeolites were pre-treated with a fresh solution of potassium chloride (KCl) 10% w/v (the regenerant) for a period of 2 h to remove any residual ammonium from the fresh media surface. Successively, 100 mL of municipal wastewater were mixed with 1 g of the media (i.e. 10 g/L), in duplicate, mixed by agitation at 150 rpm for a period of 8 h using the orbital shaker SSL1 (STUART, UK). The average initial concentration was 12.1 ± 0.3 mg NH_4_^+^-N/L (0.7 meq NH_4_^+^-N/L). At the end of each cycle, the media were regenerated with a fresh regenerant solution for a period of 2 h for a total of 10 cycles. The ammonium exchange capacity (AEC) in wastewater was calculated as in Eq. (2) while the regeneration capacity (Q_reg_) was calculated as in Eq. (3) (adapted from You et al*.*^36^):

3 $${Q}_{reg}=\frac{\left[{C}_{r.i}-{C}_{r.f}\right]*{V}_{r. treated}}{M}$$

where, *Q*_*reg*_ is the capacity of regeneration (meq NH_4_^+^-N/g media); *C*_*r.i*_ and *C*_*r.f*_ are the concentration of ammonium in the regenerant at the beginning and at the end of each cycle (meq NH_4_^+^-N/g media); *V*_*r.treated*_ is the volume of regenerant used (L); *M* is the same as in Eq. (2).

### Attrition and resistance to compression tests

For the attrition tests, 3 g of fresh Zeolite were mixed at 200 rpm (in accordance to literature^37,38^ with 300 mL of deionized water for a period of 24 h using the orbital shaker SSL1 (STUART, UK). Samples were taken at regular intervals (each 15 min during the first hour of treatment and each 2 h from 2 to 24 h of treatment), after 1 min of settling.

The media resistance to compression was measured using the system Instron 5,965 (Instron, UK). Thirty beads of each zeolite were singularly positioned in between the compressing disks of the system and subjected to an increasing load (measured in Newton, N) until breakage. The force applied at breaking point was registered with an accuracy of 0.5%. Measurements were performed on fresh media.

### Ion exchange demonstration scale plant

Zeolite-N and Zeolite6 were separately tested for ammonium removal in an ion exchange demonstration scale plant (IEX-D) (Table 2) treating 10 m^3^/day of municipal wastewater, with average ammonium concentrations of 13.6 ± 4.6 mg NH_4_-N/L (0.8 ± 0.3 meq NH_4_-N/L). Firstly, a column with an internal diameter of 307 mm and height of 1566 mm was filled with 69L (78 kg) of Zeolite-N. The media was first backwashed for 30 min to remove any fine particle left over from manufacturing. The wastewater was fed in down-flow operation at an empty bed contact time (EBCT) of 10 min. After media saturation, regeneration was completed using 10 bed volumes of 10% potassium chloride (KCl) that were passed through the column in up-flow operation for 2 h. At the end of the regeneration, the column was drained and the KCl was collected and stored for the next regeneration. The media was backwashed with tap water for 30 min (flow rate 500 L/h) at the end of each cycle to remove any residual solids.

Successively, a column with internal diameter of 217 mm and height 1577 mm was filled with 35L (24 kg) of Zeolite6 (Table 2). After an initial backwash, the wastewater was fed at an EBCT of 5 min. The same operation conditions as for Zeolite-N were used.

For both media, the ammonium was measured at the inlet and outlet of the columns and in the regenerant. The AEC and Q_reg_ were obtained as in Eqs. (2) and (3), respectively. A sample of both media was analysed at the optical microscope (Optech Microscopes Ltd) for the fresh media and for the media taken from the IEX-D columns after 7 and 3 cycles for Zeolite-N and Zeolite6, respectively and resistance to attrition test were completed (“Attrition and resistance to compression tests” section). The experiment was conducted in triplicate.

### Physico-chemical and statistical analysis

Turbidity was measured using the 2100 N Turbidimeter (HACH, UK) in accordance with the EPA 180.1 method^39^. For the characterization of the wastewater, chemical oxygen demand, calcium and potassium were analysed using Spectroquant cell tests; ammonium and phosphorus were analysed using the Smartchem200 (AMS Alliance, France). The analysis of the pH was performed using a pH meter (Jenway 3,510 pH and conductivity meter, Camlab, UK).

Statistical analyses were performed using the JMP software (SAS Institute) to identify statistical difference between the capacity of adsorption considering the solution treated (municipal wastewater and synthetic solution) and the media (natural and synthetic zeolite). The JMP tool was also used to detect any statistical difference in the ion exchange capacities of the media comparing the first and tenth cycle of batch test.
